# *In silico* Identification of IgE-Binding Epitopes of Osmotin Protein

**DOI:** 10.1371/journal.pone.0054755

**Published:** 2013-01-18

**Authors:** Prerna Sharma, Shailendra Nath Gaur, Naveen Arora

**Affiliations:** 1 Allergy and Immunology Section, CSIR-Institute of Genomics and Integrative Biology, Delhi, India; 2 Department of Respiratory Medicine, Vallabhbhai Patel Chest Institute, University of Delhi, Delhi, India; Kyushu Institute of Technology, Japan

## Abstract

The identification of B-cell epitopes is an important step to study the antigen- antibody interactions for diagnosis and therapy. The present study aimed to identify B- cell epitopes of osmotin using bioinformatic tools and further modify these regions to study the allergenic property. B-cell epitopes were predicted based on amino acid physicochemical properties. Three single point mutations M1, M2, and M3 and a multiple point mutant (M123) were selected to disrupt the IgE binding. These mutants were cloned, expressed and proteins purified to homogeneity. The IgE binding of the purified proteins was evaluated by ELISA and ELISA inhibition with patients' sera. Three regions of osmotin M1 (57–70 aa), M2 (72–85 aa) and M3 (147–165 aa) were identified as potential antibody recognition sites using *in silico* tools. The sequence similarity search of the predicted epitopes of osmotin using Structural Database of Allergenic proteins (SDAP) showed similarity with known allergens from tomato, kiwifruit, bell pepper, apple, mountain cedar and cypress. Mutants M1, M2 and M3 showed up to 72%, 60% and 76% reduction, respectively in IgE binding whereas M123 showed up to 90% reduction with patients' sera. The immunoblot of M123 mutant showed 40% reduction in spot density as compared to osmotin. All mutants showed decreased inhibition potency with M123 exhibiting lowest potency of 32% with osmotin positive pooled patients' sera. The three B- cell epitopes of osmotin predicted by *in silico* method correlated with the experimental approach. The mutant M123 showed a reduction of 90% in IgE binding. The present method may be employed for prediction of B- cell epitopes of allergenic proteins.

## Introduction

The prevalence of food allergy is increasing worldwide and as many as 8% of children and 2% of adults are affected in western countries [1]. Primary treatment of food allergy is strict avoidance of the diets containing food allergens. Recently new strategies have been designed to reduce allergic reactions [2]. Specific immunotherapy is not used to treat food allergies because of the risk of severe side effects, including anaphylactic shock [3]. Several studies have shown the development of hypoallergenic molecules with reduced or no allergenic potential, which may be useful for specific immunotherapy [4], [5].

The knowledge of the B-cell epitopes can be applied in diagnosis, therapy or development of effective vaccines for immunotherapy [6]. In case of food allergens, digestion/heating may lead to loss of the structure of the protein thereby rendering the conformational epitopes inactive. Thus sequential epitopes play an important role for diagnosis of food allergy. Moreover, it is difficult to study the conformational epitopes of allergens as considerable efforts are required for validation [7]. Epitopes can be identified by many methods but computational tools provide a promising and rapid alternative [8]. These epitopes may be modified to reduce allergenicity of an allergen. These variants can be produced by disruption of IgE binding epitopes either by chemical modification [5] or genetic engineering [9]. The key requirement is to identify IgE-binding epitopes on food allergens. The recombinant allergens have similar structure and immunologic properties as that of native counterparts, hence these can be modified to reduce the allergenicity while maintaining important immunologic properties [10]. Earlier studies on recombinant food allergens have demonstrated reduced allergenicity of shrimp [4], peanut allergen [11], apple [12], olive [13], etc.

Osmotin is a stress-responsive multifunctional protein from tobacco (*Nicotiana tabacum*). It accumulates in cells on osmotic stress adaptation and provides osmotolerance to plants [14]. It belongs to pathogenesis related-5 (PR-5) protein family and exhibits antifungal activity as it permeabilizes the plasma membrane and dissipates the membrane pH gradient of the fungal species [15]. These two properties of osmotin have been exploited for developing transgenic crops such as tobacco [16], strawberry [17], wheat [18], cotton [19], tomato [20], and mulberry [21]. In our previous study we had identified osmotin as a potential allergen using bioinformatic and immunobiochemical methods [22]. In the present study we identified sequential epitopes of osmotin by *in silico* approach. The potential IgE binding residues in these epitopes were mutated to reduce allergenicity of osmotin.

## Materials and Methods

### Ethics statement

The study protocol was approved by human ethics committee of the Institute of Genomics and Integrative Biology, Delhi. Informed written consent was obtained from the patients and healthy subjects that participated in the study. The study did not involve any minors/children.

### B-cell epitope prediction

B-cell epitopes are classified as either continuous or discontinuous. A number of algorithms have been developed for predicting the continuous B-cell epitopes based on physico-chemical properties like hydrophilicity (Parker method), flexibility (Karplus and Schulz method), accessibility (Emini method) and turns (Pellequer method) [23]. The prediction methods of the linear or continuous epitopes have improved over the years. The linear B- cell epitopes were predicted by different servers namely, BcePred (based on a combination of physico-chemical properties), BepiPred 1.0b (based on hydorophilicity scale with a Hidden Markov Model), ABCpred (uses artificial neural networks), and BCPred (uses support vector machine) [23]. DNASTAR Lasergene version 7.2. (DNASTAR Inc., Madison, WI, USA) was also used which uses amino acid propensity scales of Hopp -Woods (hydrophilicity), Emini (Surface probability), Jameson-Wolf (antigenic index) and Karplus-Schulz (flexibility). The epitopes identified frequently by most of the tools were selected.

### Site directed mutagenesis of osmotin

Osmotin was mutated at three residues to generate three single point mutants M1, M2 and M3. A fourth mutant M123 with all three point mutations was also generated. Mutagenesis was performed using oligonucleotide primers and mutation-specific oligonucleotide primers accommodating each mutation. Internal amino acid substitution in osmotin was performed by a three step PCR reaction. The first two steps of PCR reaction was carried out with an internal mutant primer and one of the flanking primers. Final products of these two steps were purified from agarose gel, mixed and amplified using flanking primers. The fourth mutant with all three point mutations was generated step wise using mutant M2 as template. The mutation 1 was generated using forward primer 5′ATCAATGCGCCACGATTTACTAAAAAG3′ and reverse primer 5′CATTTTAGTAAATCGTGGCGCATTG3′, mutant 2 was generated using forward primer 5′ATGCTGCTGGTAGGTTTACGTGCCAA3′ and reverse primer 5′TTTGGCACGTAAA CCTACCAGCAGCA3′, mutant 3 using forward primer 5′ACGGCTAATATATTCGGCGAATGTCC3′ and reverse primer 5′GGACATTCGCCGAATATATTAGCCGTAC3′ and the flanking primers 5′GAATTGAATTCGATGGGCAACTTGAGATCTTCTTTTG3′with *EcoR I* site and 5′GCTCGAG CTCCTTAGCCACTTCATCACTTCCAG 3′ with *Xho I* site. The PCR products were digested with *EcoRI* and *XhoI* enzymes, subcloned in pET22b+ vector and sequenced to confirm the mutations. Constructs containing the mutations in the epitopes were used for the expression of fully modified recombinant osmotin protein mutants.

### Expression and purification of mutants

The constructs of M1, M2, M3 and M123 mutant sequences in pET22b+ vector were transformed into BL-21 *Escherichia coli* cells. The cells were grown till 0.6 O.D., induced with 1 mM isopropyl-beta-D-thiogalactopyranoside for 3 h, harvested and the pellet was suspended in binding buffer containing 50 mM NaH_2_PO_4_, 300 mM NaCl, 10 mM imidazole (pH 8.0), sonicated and centrifuged. The supernatant was incubated for 2 h with 2 ml Ni-NTA equilibrated with binding buffer. The column was washed with buffer containing 20 mM imidazole, 300 mM NaCl and 50 Mm NaH_2_PO_4_ (pH 8.0) and bound proteins were eluted using buffer containing 250 mM imidazole, 300 mM NaCl and 50 mM NaH_2_PO_4_. The purified mutant proteins were resolved on 12% SDS-PAGE, transferred to nitrocellulose membrane and probed with osmotin positive patients' pooled sera. Densitometry of the immunoblot was performed by Gene Tools software from SynGene (Synoptics Ltd, Cambridge, UK).

### Circular dichroism (CD) spectroscopy

Osmotin protein and its mutant M123 were dialyzed against 10 mM KH_2_PO_4_/K_2_HPO_4_ buffer (pH 7.4). Circular dichroism spectroscopy was performed on a J-815 S spectropolarimeter (Jasco) with constant nitrogen flushing at 25°C. Wavelength was analysed with a step width of 0.2 nm and a band width of 1 nm. The spectral range was 200–240 nm at a scan speed of 50 nm/min. The final spectra were corrected by subtracting the corresponding baseline spectrum obtained under identical conditions. Average of three scans was taken and the mean residue molar ellipticity was calculated.

### Sera collection

The patients of allergic rhinitis and asthma aged 15–50 years were skin prick tested with various aeroallergens and food extracts to detect sensitization. The guidelines of the American Thoracic Society were followed for diagnosis of asthma [24]. The patients having any two of the symptoms, viz. sneezing, rhinorrhea, nasal blockage, postnasal drip, etc. for most of the time since the last two years were diagnosed as rhinitis. Blood samples were collected from patients showing marked positive skin reactions to different food extracts and healthy subjects at the outpatient department, V.P. Chest Institute, Delhi, a referral chest hospital.

### Specific IgE estimation

Specific IgE binding for the mutants and osmotin protein was determined by ELISA. Briefly the proteins were incubated in carbonate buffer overnight at 4°C in a microtitre plate (Nunc, Roskilde, Denmark). After washing with PBS– Tween 20 (0.05%), the nonspecific sites were blocked with 3% defatted milk for 3 h at 37°C. The plate was washed again and incubated with 100 µl diluted (1∶10 v/v) sera of food allergic patients which were positive to osmotin, overnight at 4°C. Normal human sera (n = 4) were used as negative control. The plate was washed and incubated with antihuman IgE-horse radish peroxidase (1∶1000 v/v, Sigma) for 3 h at 37°C. The plate was washed with PBS- Tween 20 and followed by PBS and colour was developed with orthophenylene diamine in citrate – phosphate buffer, pH 5.0. The reaction was stopped after 30 min by adding 5 N H_2_SO_4_ and the absorbance was read at 492 nm.

### ELISA inhibition

ELISA inhibition with the mutants was performed to determine the allergenic potency and relative ability of the mutants for IgE binding. The microtitre plate was coated with purified osmotin protein and blocked with 3% defatted milk. Further it was incubated with pre- incubated mixture of 1∶10 v/v osmotin positive pooled patients' sera (n = 5) and graded amounts of osmotin and each of the purified mutants i.e. 0.1, 1, 10, 100 and 1000 ng as inhibitor. Sera of non-allergic subjects were used as control. The remaining steps were the same as followed for ELISA. Inhibition was presented as percentage decrease in absorbance of sample with inhibitor to that of sample without inhibitor and allergenic potency was defined by establishing the concentration of protein required for 50% inhibition.

### Statistical analysis

Statistical analysis of results was done by using GraphPad Prism and GraphPad Instat software (GraphPad Software, San Diego, CA, USA). The statistically significant difference was determined using one way ANOVA followed by Bonferroni comparison test between osmotin and the mutant M123 challenged mice groups. The p value <0.05 was considered as significant.

## Results

### Mapping of IgE-binding epitopes

**Figure 1:**
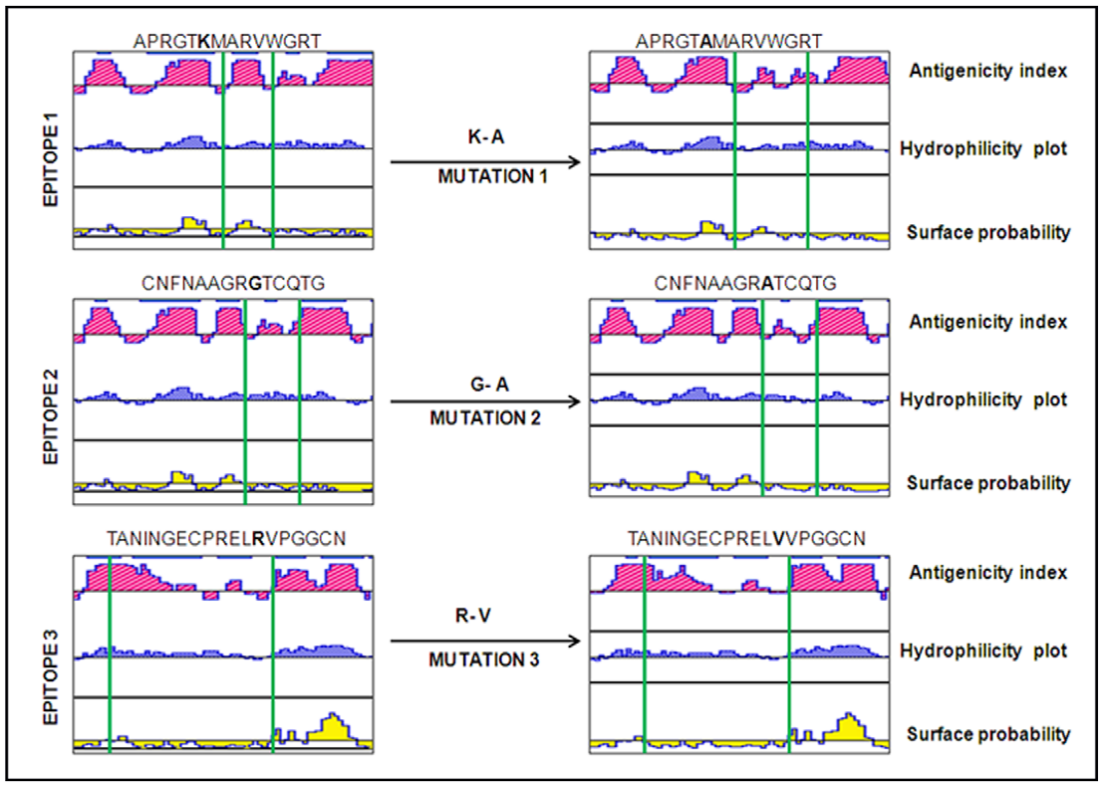
Comparative analysis of antigenic index of osmotin epitopes and its mutants by DNAstar protean system. The region between vertical lines show the three regions mutated by site directed mutagenesis. Mutation in epitope regions shows reduction in antigenic index in DNAstar program.

IgE binding regions were identified by sequence based tools which fall in common regions of the sequence map. Three stretches of amino acids 57–70, 72–85 and 147–165 recognized by at least four tools were predicted as B- cell epitopes. Residues 60 (Gly → Phe), 80 (Gly → Phe) and 151(Asn → Phe) in each epitope were selected for substitution mutation. These epitopes on mutation using *in silico* approach showed reduction in the antigenic index in DNAstar program (Fig. 1). In Structural Database of Allergenic Proteins (SDAP; 737 sequences; http://fermi.utmb.edu/SDAP/), these epitopes showed similarity with tomato allergen Lyc (NP24), kiwi fruit allergen (Act c 2), cypress allergen (Cup s 3.0102), bell pepper allergen (Cap a 1), apple allergen (Mal d 2) and mountain cedar allergen (Jun a 3) with a *PD* (property distance) value of less than 8.5 (Table 1).

**Table 1:** Peptide similarity search of the three predicted epitopes of osmotin with known allergens in SDAP database.

S. No.	Predicted osmotin Epitopes	Allergen	*PD* Sequence Similarity Index	Matching region
1	APRGTKMARVWGRT	Lyc e NP24	0.39	**APRGTKMAR**i**WGRT**
		Cap a 1	2.01	**AP**p**GTKMAR**i**WGRT**
		Cap a 1w	3.47	**AP**p**GT**a**MAR**i**WGRT**
		Act c 2	7.31	pgag**TK**g**ARVW**p**RT**
2	CNFNAAGRGTCQTG	Lyc e NP24	0.00	**CNFNAAGRGTCQTG**
		Act c 2	2.92	**CNF**dg**AGRG**k**CQTG**
		Jun a 3	3.26	**C**t**F**d**A**s**G**k**GsCQTG**
		Cap a 1	3.39	**CNF**dgA**GRG**w**CQTG**
		Cup s 3.0101	4.34	**CtF**d**A**s**G**g**G**s**C**rs**G**
		Cup a 3	4.34	**C**t**F**d**A**s**G**k**G**s**C**rs**G**
		Mus a 4.0101	4.49	CSFDGSGRGRCQTG
		Mal d 2	7.08	**C**std**AAG**kft**C**e**T**a
3	TANINGECPRELRVPGGCN	Lyc e NP24	1.21	**TANINGECPR**a**L**k**VPGGCN**
		Cap a 1w	2.42	**TANINGECP**gs**LRVPGGCN**
		Act c 2	2.46	**TA**d**ING**q**CP**n**ELR**a**PGGCN**
		Cap a 1	3.24	v**ANINGECP**gs**LRVPGGCN**
		Mus a 4.0101	5.07	aad**INGqCP**ga**L**ka**PGGCN**
		Cup a 3	5.73	kad**IN**av**CP**s**EL**k**V**d**GGCN**
		Jun a 3	5.79	kad**IN**av**CP**s**EL**k**V**d**GGCN**

The table shows similarity of the predicted B- cell epitopes of osmotin with peptides of known allergens with a PD value less than 8.5 in SDAP database. Matching amino acids are depicted in bold and upper case. Unmatched amino acids in lower case.

### Expression and purification of the mutants

**Figure 2:**
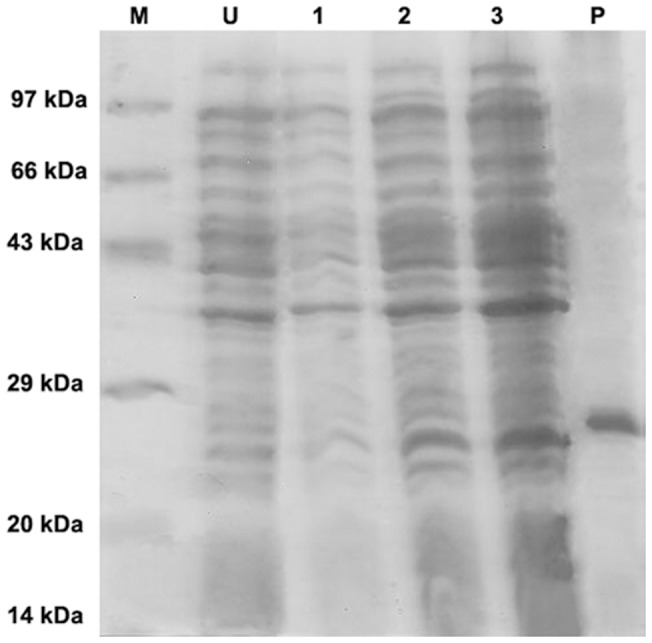
SDS-PAGE analysis of protein expression in culture. The uninduced and induced stages of expression of osmotin protein was resolved on 12% gel and stained with Coomassie brilliant blue. Lane M was loaded with Molecular weight marker. Lane U uninduced protein expression. Lane 1, 2, 3 shows expression after induction for 1 h, 2 h, 3 h respectively and lane P represents purified protein.

**Figure 3:**
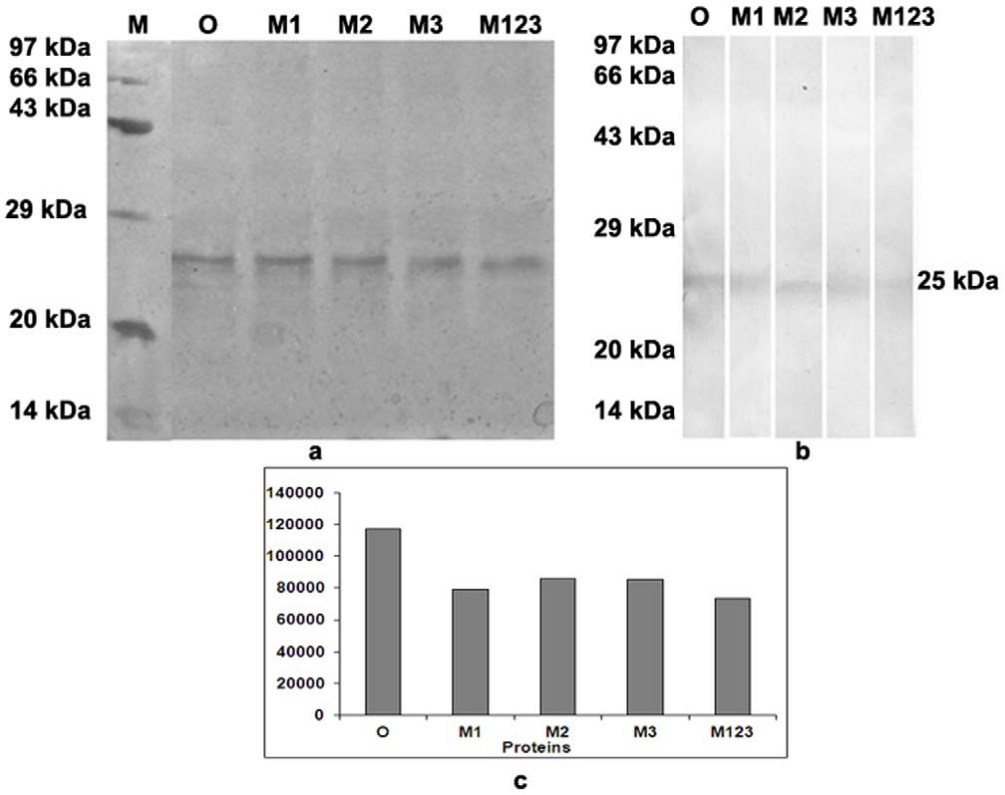
SDS-PAGE profile of purified osmotin and the three mutants. (a) The proteins were resolved on 12% gel and stained with Coomassie brilliant blue. (b) SDS-PAGE resolved protein was transferred on to nitrocellulose membrane and probed with osmotin positive patients' pooled sera. Lane M was loaded with Molecular weight marker. Lanes O, M1, M2, M3, M123 were loaded with osmotin and the mutants respectively. (c) Graph showing densitometric scan of spots of the blot.

Four osmotin mutants M1, M2, M3 and M123 proteins were expressed in *E. coli* and purified as His-tag proteins. The yield obtained of these proteins were 2.5 mg/L, 2 mg/L, 2 mg/L, 3 mg/L of the culture. The protein expression in the culture after induction at 1h, 2 h and 3 h with IPTG was visualized by SDS-PAGE (Fig. 2). The purified recombinant proteins were resolved on SDS-PAGE (Fig. 3a) and reacted with osmotin positive pooled patients' sera on immunoblot (Fig. 3b). The intensity of spots on the blot was measured by densitometric scan and osmotin was set as a reference point. The mutant M123 showed 40% reduction in the spot density followed by M1, M3 and M2 as compared to osmotin (Figure 3c).

### Circular dichroism analysis of purified recombinant osmotin and mutant M123

**Figure 4:**
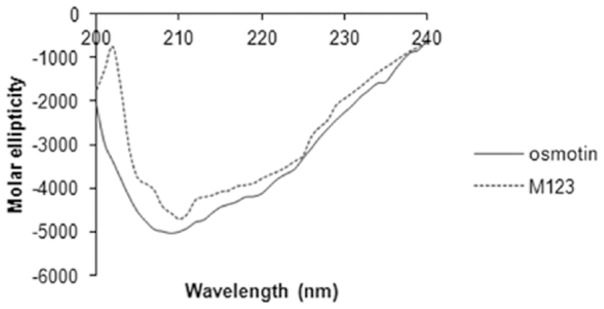
Circular dichroism analysis. Far-UV CD analysis of purified osmotin protein and M123 mutant. Results are expressed as mean residue ellipticity (y-axis) at a given wavelength (x-axis).

Figure 4 shows the comparison of the osmotin protein and M123 mutant by circular dichroism analysis. The far-UV spectra showed similar characteristics. Minima were obtained at 208 nm, 218 nm and 222 nm. It shows the presence of both α- helices and β- sheets in the secondary structure of the osmotin protein. The CD spectrum of the M123 mutant was similar to osmotin indicating that the mutant has probably adopted the same folded state.

### IgE binding of the mutants with osmotin positive patients' sera

**Figure 5:**
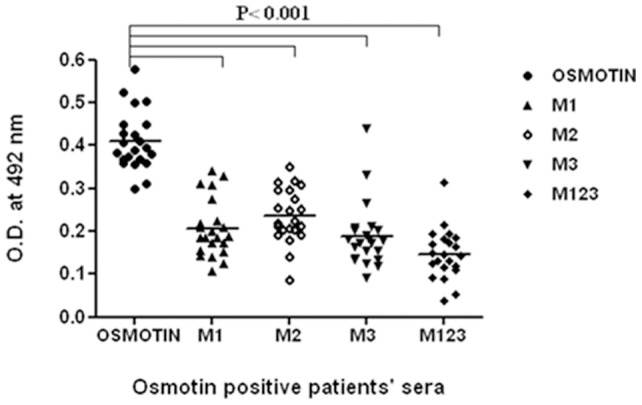
IgE affinity of osmotin, M1, M2, M3, M123 with food allergic patients' sera (n = 22). Specific IgE values against the respective proteins in patients' sera by ELISA. Specific IgE values ≥3 times of control were taken as cutoff for the positive results. (─) median value. Statistical difference in IgE binding among osmotin and the mutants is p<0.001 was considered statistically significant.

IgE-binding of the four mutants was compared with that of osmotin by ELISA. The mutants showed a reduction in specific IgE binding between 25%–72% (M1), 22%–60% (M2), 43%–76% (M3) and 50%–90% (M123) as compared to osmotin (Fig. 5). All the mutants showed significant reduction in IgE binding as compared to osmotin (p<0.001) with mutant M123 having maximum reduction.

### Potency of osmotin mutants

**Figure 6:**
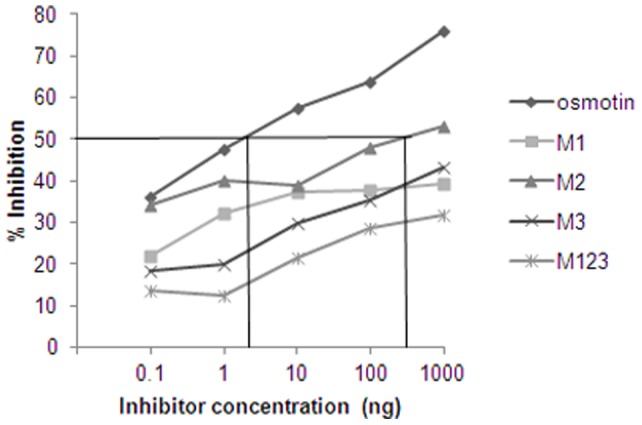
IgE reactivity of osmotin, M1, M2, M3 and M123. For IgE inhibition, osmotin positive patients' pooled sera were incubated with 0.1–1000 ng of osmotin protein and mutants as inhibitor. The assay was carried out with purified osmotin protein coated on microtitre plate. Competition of IgE binding to osmotin on the solid phase was quantified by ELISA. The (EC_50_) value for Osmotin is 4 ng and for M2 is 500 ng.

IgE-binding and immunoblot of the mutants were further substantiated by ELISA inhibition assay. Pooled osmotin positive patients' sera preincubated with different amounts of the four mutants were added to ELISA plate coated with osmotin. Here, wild-type osmotin gave 66% inhibition at 1000 ng of protein. In contrast, all mutants showed decreased ability to inhibit IgE binding. M1, M2, M3 and M123 reached a maximum inhibition of 39%, 53%, 43% and 32% respectively at 1000 ng/ml inhibitor concentrations. Osmotin required 4 ng of protein to reach the EC_50_. Only M2 showed the inhibition with an EC_50_ of 500 ng whereas the other mutants did not reach the EC_50_ value (Fig. 6).

## Discussion

The knowledge of epitopes is a prerequisite for understanding mechanism of antigen antibody interactions. Various methods like X-ray crystallography, enzymatic fragmentation, synthetic overlapping peptides, site-directed mutagenesis and phage display may be used to determine B- cell epitopes. However, these approaches are time consuming, exhaustive and require many experiments. Hence, we used computational methods to predict the location of B- cell epitopes. Earlier studies have shown the use of bioinformatic approach to predict eptiopes which correlated well with the experimental approach [8], [25]. In the present study, three IgE binding regions of osmotin were identified and validated by *in vitro* experiments. Further, residues in these regions were mutated to study the allergenic properties.

Linear epitopes are more relevant as compared to conformational epitopes for food allergens as these allergens are encountered by the immune system only after they are subjected to digestion/heating [26]. Further it is difficult to validate computationally predicted epitopes except by cocrystallographic approach [7]. Conformational epitopes are preferred in case of globular allergens such as aeroallergens or food allergens involved in oral allergy syndrome [27]. It has been demonstrated that many food allergens like Ara h 1, Ara h 2, Ara h 3 [28], shrimp allergen Pen a 1 [29], hen's egg ovomucoid [30] and milk allergen [31] have linear epitopes that binds to IgE. Many algorithms have been developed to predict linear epitopes on a protein sequence based on propensity values of amino acid properties of hydrophilicity, antigenicity, segmental mobility, flexibility and accessibility [27]. In our study, we selected three linear epitopes commonly recognized by most of the sequence based tools. The three epitopes predicted comprise approximately 50% hydrophobic residues and 25% charged residues. The SDAP sequence similarity search of the predicted epitopes of osmotin showed similarity with known allergens which are members of the PR-5 protein family. Maleki et al., 2011 reported that peptides with a *PD* value similarity (<8.5) to known IgE epitopes could contribute to IgE binding and cross-reactivity [32]. This indicates that osmotin may share cross reactive epitopes with other similar proteins. In our earlier study osmotin showed IgE binding with sera of tomato and apple allergic patients confirming the predicted epitopes [22].

In the present study, three single point mutants (M1, M2 and M3) and a triple point mutant, M123 was subcloned and protein purified to homogeneity. These were developed by mutating a single residue in each of the three IgE binding predicted areas. Previous study has shown that single point allergen mutants do not lead to significant changes in protein structure but reduces allergenicity [33]. Mutational analysis of Pen a 1 showed that at least three substitutions per epitope were necessary to abolish the IgE Ab reactivity in >99% of the mutated epitopes [4]. In our study, all the mutants of osmotin showed reduction in IgE binding with patients' sera. The pattern of IgE binding of the mutants was not similar among all the patients suggesting the contribution was not same for all the mutations. However, the triple mutant of osmotin showed maximum reduction of up to 90% in the IgE binding as compared to osmotin. The reduction in IgE binding may be attributed to the phenylalanine substitution that is non reactive and tend to bury in hydrophobic cores of the protein molecule [34].

The *Brassica* pollen allergen Bra r 1 showed 74% and 94% inhibition of IgE binding with two patients' sera respectively at the highest inhibitor concentration. The double mutant of Bra r 1 showed 13% and 19% inhibition indicating reduced affinity of the IgE antibodies [35]. Peach allergen, Pru p 3 mutated at single position showed no change in IgE binding pattern whereas the triple mutant showed a 5 times reduction in IgE binding and inhibition potency [36]. Allergic response is elicited due to the multiple epitopes on an allergen, therefore multiple mutations are required to inhibit IgE binding completely. In the present study, triple mutant M123 of osmotin showed up to 90% reduction as compared to osmotin whereas single point mutants showed reduction of up to 76% in IgE binding. The circular dichroism spectra of the recombinant osmotin and M123 mutant showed that the secondary structure was not affected by three substitutions.

The potential of introduced foreign or engineered proteins in genetically modified crops to cause allergy has been of concern worldwide [37]. Many food allergens have been modified to generate the hypoallergenic mutants that showed reduced IgE binding. Osmotin has been shown as a potential allergen using *in silico* and *in vitro* studies [22]. It is still being used for developing transgenic crops owing to its properties of osmotic stress tolerance and antifungal activity. Therefore, genetically modified foods containing osmotin may not get clearance from regulatory bodies. Osmotin with no IgE binding having biological activity may be employed in developing genetically modified crops.

Specific immunotherapy against allergic disorders requires frequent injections of allergen extract to slowly build up the maintenance dose [38]. Allergen specific immunotherapy with purified protein has shown potential but may cause anaphylactic reactions. This can be prevented by modifying the IgE binding epitopes of the protein. In the present study IgE binding linear epitopes of osmotin were modified to have a hypoallergenic. These mutant proteins will have potential for immunotherapy.

In conclusion, three B- cell epitopes of osmotin were predicted by *in silico* method that correlated well with the experimental approach. Osmotin mutant M123 showed a reduction of 90% in IgE binding. The present method may be employed for prediction of B- cell epitopes of allergenic proteins.
